# The science behind the lifesum app: an intervention design analysis

**DOI:** 10.1038/s41598-025-97852-0

**Published:** 2025-04-23

**Authors:** Signe Svanfeldt, Chris Seth, Marcus Gners, Andreas Blomqvist

**Affiliations:** 1 Lifesum AB, Repslagargatan 17B, Stockholm, 118 46 Sweden; 2 Department of Health, Medicine and Caring Sciences, Linköping University, Linköping, 58183 Sweden

**Keywords:** Behaviour change, App, Wellness, Healthy eating, Intervention development, Macro-nutrient balance, Software, Nutrition

## Abstract

Wellness is an increasingly important part of public health and can prevent both disease and death. Diet and nutrition are important factors that contribute to wellness and predict health outcomes. Adhering to healthy diets is notoriously difficult for many, and some support is often required. Increasingly, that support may be found in the shape of an app in a smartphone. One such app is Lifesum, with some 65 million users worldwide. Lifesum adopts a more holistic approach to nutrition and well-being, and adopted an evidence-based approach to its development. The aim of this study was to describe the scientific, theoretical basis for the Lifesum app and contribute to advancing science in the field of wellness app development. This was an intervention design analysis, designed to describe the theoretical model and intervention theory used to create the Lifesum app in its current embodiment. A pragmatic theoretical model describing behaviour change in the context of healthy eating was devised based on findings in literature. Factors that drive unhealthy eating behaviours, but that were malleable and whose mechanisms of change were feasible to implement, were identified and used to form an intervention theory. The theoretical model and the intervention theory could then guide the implementation of the Lifesum app, illustrated by a logic model. The theoretical model emphasizes personal goal-attainment and motivation as keys to establishing and maintaining healthy eating behaviours, with proximal outcomes being nutrition knowledge, mindfulness about eating and macro-nutrient balance. Nutrition knowledge is achieved through the provision of nutrition information from a vast database on food items, easily available. Continuous feedback on food choices made will enhance this knowledge and a greater awareness of the impact of nutrition on health remains desirable. A more mindful disposition regarding foods is achieved through support in terms of tracking food intake continuously, as well as recommending meals or recipes. After collecting user preferences on health status, biometrics and goals, these meal plans and recipes can be made to offer the optimal macro-nutrient distribution for each individual user. A theoretical model for diet-related behavior change was developed and key dietary issues were identified, outlining mechanisms for positive impact. These insights informed a mechanistic description of the Lifesum app, providing a foundation for future research on intervention outcomes.

## Introduction

What is wellness and does it matter? The answer may not be as obvious as one would think, and there is no one uniform definition^1^, although it clearly matters to many as the wellness market is estimated to be worth around $1.5 trillion^2^. Since there is no widely accepted definition of wellness, the definition of health is most often used, which is “a state of complete physical, mental, and social well-being and not merely the absence of disease or infirmity”^3^. That wellness matters to the individual is obvious, but it also matters on societal levels because it increases labour productivity, decreases risks of morbidity and mortality and lowers health-care costs, i.e. is an important concept for public health^3,4^.

Public health is important and influences public policies around the world. Many governments aim to mitigate health risks by addressing diets and nutritional flaws^5^ and the burden of nutrition-related disease continues to rise worldwide^6^. There doesn’t seem to be a one-size fit all solution when it comes to dietary advice, because there are multiple feasible paths, all of which are efficacious for some and marred with issues for others^7^. Since there certainly aren’t enough diet experts and coaches around for every person to get the type of individual support these findings suggest is needed, we may have to turn to technology for a solution.

Smartphones are ubiquitous these days, and as a consequence there are millions of users that are exposed to some sort of wellness app through their smartphone^8^ and there are hundreds of thousands of health-related apps available^9^. These are not mere pastime trinkets, but are expected by the World Health Organization to play an important role in managing health^10^, and although the efficacy is still somewhat debatable there are clear indications of usefulness in recent systematic reviews^11,12^.

One app in the wellness field is Lifesum, designed in Sweden originally in 2013, to help users improve their overall health through personalized nutrition and lifestyle recommendations, currently with some 65 million users around the world.There are vast numbers of apps out there, which makes it increasingly hard for users to know where to turn and to assess quality. While plenty of apps available focus on diet and counting calories as both means and outcomes^13^, the new Lifesum app reported on herein recognizes that healthy eating is much more than that. The algorithms implemented are unique and also incorporates gamification, which is known to improve adherence^14^. Furthermore, Lifesum incorporates other features revolving around personalization of recommendation based on goals, diets (such as mediterranean, vegan, high protein etc.) which will be explored in depth in this study. There is evidence to suggest that more blunt nutrition apps can have harmful effects and precipitate eating disorders^15^. To mitigate such risks, there are built-int controls, e.g. body-mass index (BMI) based, which prevents unhealthy use of the app. It has been shown that in order to achieve adherence to gamified and incentivized mobile apps, an evidence-based design is advisable^16^. In lieu of this, we sought to contribute to improving the body of science pertaining to this field of wellness-apps, by analysing the development and design of the Lifesum app from a scientific perspective, in an evidence-based manner.

To summarize the introduction; there is substantial evidence to support the importance of good nutrition, there is also evidence to support the concept of modifying behaviour using novel technology. However, high attrition rates are common, and focus on maintenance, i.e. sustained behaviour change is key. There is a lack of detailed, scientific descriptions of evidence-based nutrition apps.

## Aim

The aim of this study was to describe the evidence-based theoretical basis for the development and design of the Lifesum app.

## Methods

This was an intervention design analysis, which is a structured approach to evaluating and optimizing an intervention before, during, or after implementation. It typically consists of problem identification and/or needs assessment, an intervention design and depending on its level of maturity, potentially also implementation- and evaluation analyses. The methods section of this manuscript will describe the approaches and principles used to define the Lifesum intervention, covering the theoretical framework and theories applied. The paradigm that the intervention was to rest on is captured in these three postulates:

1. Nutrition matters for the individual’s wellbeing and health.
2. Behaviours related to nutrition can be modified using modern mobile technology.
3. The end-goal is sustained behaviour change, and maintenance of healthy eating behaviours.

We based our methods on the guidance on developing complex interventions, published by the Medical Research Council (MRC)^17^. The MRC is quite substantial, and covers everything from the spawning of a new idea to evaluating its implementation into for instance clinical practise. We decided to focus on producing what the MRC deems to be the most important constituents of a solid basis for a new intervention. These are a theoretical model, an intervention theory, a logic model and an intervention design, and together constitute the results of this study. Workshops with the design-/product teams were coupled with literature reviews to create these aforementioned outputs and sources for finding literature were Science direct, PubMed and Google scholar.

### Theoretical model

There are many published theories on behaviour change, and it can be argued that the most important are the Transtheoretical Model of Change, the Theory of Planned Behaviour, Social Cognitive Theory, the Information-Motivation-Behavioural-Skills Model, the Health belief model and self-determination theory. It is commonly recognised that new health behaviours are often not maintained^18^, and most ofteninterventions are not properly based on an appropriate theoretical framework^19,20^.

A theoretical model is generally made up of several components. One such component consists of antecedents, i.e. predictors or determinants, which are the factors or variables that influence the behaviour or outcome of interest. The other main component is the outcomes. Outcomes are the end results or behaviours that the model aims to explain or predict. Other components that constitute the model are mediators and moderators. Mediators are variables that explain the process through which antecedents affect the outcome, and are often mechanisms or pathways through which the change occurs^21^. Moderators are variables that influence the strength (or sometimes the direction) of the relationship between antecedents and outcomes^21^.

### Intervention theory

The MRC issues some of the most important guidance on the development of complex interventions^17^ and it argues for a clear description of an intervention theory which clarifies the choices made in the intervention design^22^. The purpose of the intervention theory is to mechanistically describe the theoretical relations between the intervention and its outcomes, i.e. to illustrate causality^23^. To define the intervention theory, we relied on methods described by Wight et al. in the paper “Six steps in quality intervention development”^24^, where you go through the following steps:

1. define and understand the problem at hand and its causes.
2. identify which of these that are modifiable, have the greatest scope for change and have the greatest impact.
3. describe the means by which said modification of said problem will be achieved.

These factor-action pairs listed will lead to the formulation of the intervention theory.

### Logic model and intervention design

With the intervention theory as well as the theoretical model in place, the implementation, or design, of the application/intervention can be decided on. The design is based on the theoretical model and the intervention theory and may end up using some or all the factor-action pairs identified. To illustrate which features were implemented in the app or intervention, which the end-user will ultimately be exposed to, and how these are meant to address the modifications listed in the intervention theory, a logic model was created. A logic model is a “systematic and visual way to present and share your understanding of the relationships among the resources you have to operate your program, the activities you plan, and the changes or results you hope to achieve”^25^. It is a graphical representation of the end-product of the development efforts.

## Results

### Theoretical model

We sought to find a pragmatic theoretical model to guide the development and understanding of the Lifesum intervention and its users, and an appropriate model for app driven behaviour change was developed from synthesis of published research. One systematic review of theories for behaviour change synthesized their findings into five themes that are important to maintenance of new behaviours^26^. These themes are *Maintenance motives* - Satisfaction with outcomes; *Self-regulation* - if the person is successful engaging in the new behaviour and finding ways to overcome barriers to pursue them; *Habits* - Successful self-regulation forms new habits. Cues to trigger the desired behaviour become important; *Resources* - effort needed to enact behaviour must be low (low opportunity cost); and *Environmental and social influences* - These are aspects that lower opportunity cost, such as a supportive environment.

In addition to adhering to these principles in order to achieve sustained behaviour change, the intervention developed needs to be feasible, which means it can be used by an individual as part of their daily routine or in relation to a particular activity or behaviour, and goal setting and real time feedback have been found to be the most effective strategies in securing behaviour change^27^. Furthermore, outcome expectation, efficacy expectation and testimonials all matter to ensure successful adoption of an intervention^28^. While the theoretical model captured most of these aspects, some are instead covered in the intervention theory or logic model, as presented in the results section.

**Fig. 1:**
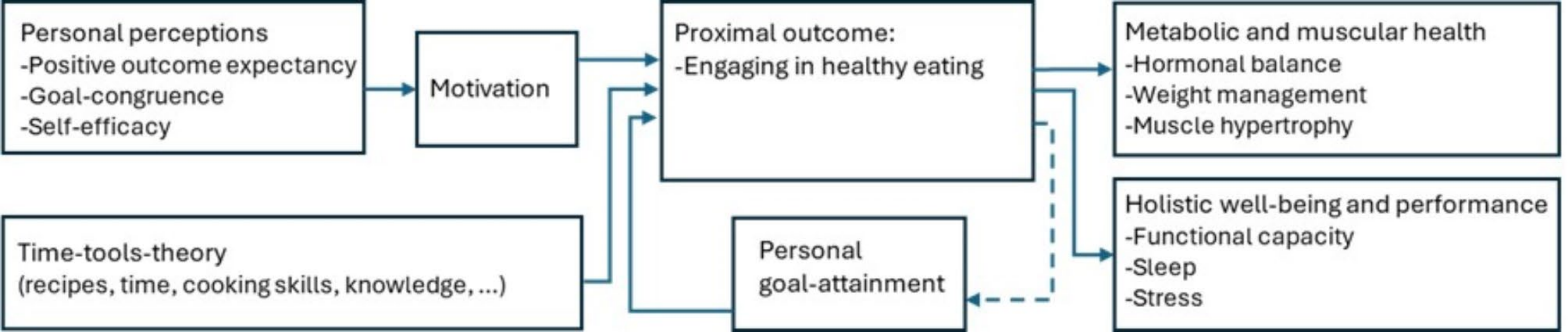
Graphical representation of the theoretical model, describing behaviour change in the context of healthy eating.

The resulting model is illustrated in Fig. 1.

### Antecedents

The first antecedent is *positive outcome expectancy*^18^. This means that the user believes that eating healthily has positive effects for them specifically. That is, it is different than a general understanding that diet matters and more relates to a conviction that certain dietary modifications will affect certain physiological and/or psychological factors in the individual adhering to the diet.

The second antecedent is *goal-congruence*^18^. It means that a particular outcome of an activity or behaviour (in this case healthy eating), is an outcome that the individual values and desires to attain.

Thirdly, we have included *self-efficacy*^18^. Self-efficacy pertains to beliefs held about one’s capability to adhere to the behaviour in question, which in this setting is eating healthily.

The fourth and final antecedent we call *time-tools-theory* and is based on the more general resources-construct, present in much behaviour change research^29^. It is a catch-all term for what practically needs to be in place for the behaviour to be actionable. A non-exhaustive list can be time to conduct the activity, access to what you need to perform it e.g. recipes, time, cooking skills, and knowledge about what to do and how to do it.

### Outcomes

The proximal outcome we defined as engaging in the desired behaviour, i.e. healthy eating. This outcome can be broken down into a plurality of attributes. We therefore went back to this outcome, once the intervention theory work was done, and let that manifest in the logic model of the designed app.

The distal outcomes we included in this model are binned in two groups (see Fig. 1). The first group, metabolic and muscular health, is made up of hormonal balance, weight loss/maintenance and muscle hypertrophy. The second, holistic well-being and performance, is functional capacity, sleep and reduced stress. Engaging in healthy eating has been shown to lead to improvements in hormonal balance^30–32^, weight management^33–35^, muscle hypertrophy^36–38^, functional capacity^39–41^, sleep^42–44^ and stress^45–47^, although it should be noted that for stress, the relationship is bi-directional.

### Mediator

We defined *motivation* as a mediator. In the context of this model, we view it as the will and drive to eat more healthily. Once these personal perceptions (inspired by Ryan et al.^18^) covered by the first three antecedents are in place, i.e. the belief that the suggested behaviour leads to a certain outcome, that this outcome is desirable and that the individual can carry out the activities required; this leads to motivation. This motivation in turn, assuming the presence of the fourth antecedent, will result in the proximal outcome.

### Moderator

A moderating effect in the theoretical model is personal goal-attainment. The sense of achieving one’s goals, we believe will increase the engagement in the behaviour in question. A user that seeks to reduce waist circumference, increase muscle mass or have more energy during the day, and experiences this, will be more diligent in executing that behaviour. We recognize that these achievements are very closely related to the listed distal outcomes. However, we make the distinction that where the distal outcomes are objective and measurable, this construct deals with the subjective experience of achievement and attaining one’s goals.

### Intervention theory

#### Identified factors

We studied the literature to find problems (or factors) relating to diet or food-consumption that in some way had detrimental effects on mental and physical well-being. These are the findings.

Poor diet quality is more often than not a consequence of not knowing how to make healthier choices^6,48–53^. These unhealthy choices in turn can contribute to reduced physical wellbeing and mental stability and increase the risk of hypertension^54,55^. Unhealthy food choices due to limited knowledge was identified as a factor.

It is common to mistake other emotions for feelings of hunger, often resulting in binge-eating^56,57^. Eating-disorders are common, with possibly as many as 100 million people affected world-wide^58^. These are especially common in Western countries and in females^59^, with binge-eating the most common type^60^. Eating-disorders can be triggered by work-related stress^61–63^, and from feelings of being isolated or lacking the protective factor of social support from co-workers when working from home^64–66^. Furthermore, people who are not actively aware of physical and emotional sensations while eating or while being in a food related environment (i.e. being mindful while eating), tend to consume more calorie dense food^67,68^. Unawareness about eating and food, as well as eating disorders were identified as two more factors.

Also, many decisions about food are automatic in nature, and most of the decisions being made are made unconsciously^69,70^. Habits and implicit perceptions were identified as a factor.

While there is no clear agreement on macro-nutrient contribution to being overweight, diets and advice about macro-nutrients matter and should be personalized^71–73^, and unbalanced macros can cause over-eating, mood- and mental disorders and negatively impact longevity^45,73–75^. Unbalanced macro-nutrients, and sub-optimal macro-nutrient distribution given a certain goal, were identified as factors.

Glucagon-like peptide-1 (GLP-1)-drugs are now indicated for treating obesity, and leads to decreased appetite, and a reduced caloric intake^76^. There is some evidence suggesting that this caloric affects the macro-nutrient balance, e.g. less reduction of carbohydrates than in protein has been reported^77^. Furthermore, since there are fewer calories consumed^77^, understanding what healthy food is in order to meet the demands of the body^78^. Again, nutrition knowledge and macro-nutrient balance emerged as factors.

To summarize, the factors we identified were; unhealthy choices due to poor knowledge, unawareness about eating and food, eating disorders, habits and implicit perceptions, and unbalanced/suboptimal macro-nutrient distribution.

#### Scientific support for the malleability of the identified factors

Knowledge provided about nutrition may lead to altered food behaviours and to healthy eating^6,48,79^, but there needs to be a behaviour change approach rather than simple information provision for efforts to be efficacious^80^.

Multiple clinical programs that strived to increase awareness have successfully employed self-monitoring^57,81–83^. Incorporated mindfulness principles have shown positive outcomes relative to eating regulation^84–86^. Mindfulness approaches can also positively influence eating disorders^68,84,86^. Avoiding simple caloric restriction to reduce feelings of being deprived is important, to not create new problems while introducing the principles of mindfulness^57,81,82,87^. An efficacious strategy to not overwhelm the user with too many goals to meet (like enough fibers, fish, protein etc.), is to instead “cloak” a plethora of advice by recommending a meal or recipe^69^.

Implicit attitudes may be affected by information but may require additional behavioural support to be efficacious^51,69,70^. Changing dietary habits is possible and may confer better benefits than medical care^50^ and interventions have good effects on inducing changes in behaviours related to nutrition and food selection^51^.

It is likely that macro-nutrient imbalance can be shifted to a more desirable state. For instance a redistribution of macros can provide increased feelings of being full (satiety)^71,88^, and since an appropriate macro-nutrient balance affects the potential to both lose fat^89^ and gain muscle^37^, motivation may be used as a catalyst to drive this change. Recipes and meal-plans provided can assure the appropriate macro-distribution, if data on the user and user preferences are collected.

### Mechanisms of action for targeting selected factors

The factors selected were the ones that there are scientifically based reasons to believe we can influence, and that have impact on the outcomes we wish to achieve. These factors, the mechanism(s) by which to affect them (causation) and what that may lead to, as well as scientific grounds for these assumptions, are listed in Table 1 (note that one action can remedy more than one factor). The intervention theory reflects these findings.

The factor-action pairs now allow us to formulate the intervention theory, which should then be used in connection with the theoretical model to design the app and its features. The intervention theory should illustrate how a certain change mechanism to affect a certain factor should be delivered^24^. We formulated the intervention theory as follows:

- Collect user preferences as to what they wish to achieve (e.g. weight loss, weight maintenance, muscle gain, vegan diet etc.). This allows us to make suggestions on what foods to eat, including recipes and meal plans to cater to these goals provided daily, which leads to control over the macro distribution and allows the user to focus on one straightforward goal (e.g. vegan or weight loss) and not generate negative feelings to being deprived of something or stressing about all the things they need to remember.

**Table 1:** Factor-action pairs identified, and supporting evidence for the claim that the action affects the factor.

Factor	Action	Evidence-based claim
Unhealthy choices due to poor knowledge Habits and implicit perceptions	Education and information about healthy eating	Information can play a primary role in food-related behaviours and is associated with choosing more healthy foods^70^ Study confirms a significant correlation between nutrition knowledge and diet quality^54^ Nutrition knowledge is a significant predictor of dietary intake^6,49,52^
Unhealthy choices due to poor knowledge Habits and implicit perceptions Reduced appetite due to GLP-1 agonist treatment	Nutrition-focused app to provide information	App users improved their objective and subjective knowledge of healthy food^90^
Unawareness about eating and food Eating disorders	Activate and engage user for each meal (tracking)	Making conscious food choices, and being aware of the present moment when one is eating leads to mindful eating^85^ External factors (like presence of an app), can influence the natural way to act about foods, and for instance prevent overeating^81^ Engage in self-monitoring prevents overeating in spite of calorie restriction^81^ Self-monitoring improves self-regulation, which is useful in achieving healthy dietary restraint^82^
Unawareness about eating and food Eating disorders	Provide goal-oriented meal plans, and make them appealing in order to limit feelings of deprivation	If a person focuses on one goal (i.e. protein rich food, more fibres, more fish or leafy greens etc.) instead of being overwhelmed by grand and intangible concepts such as “eat healthy”, dieters may be more successful^69^ The cognitive effort required to deprive oneself of food may cause eating disorders^87^ Limiting perceived deprivation inhibits weight gain^82^
Unbalanced or suboptimal macro-nutrient distribution	Collect user input (preferences and biometrics)	A certain macro-distribution makes it easier to unconsciously reduce spontaneous energy intake^89^ Diets and advice need to be personalized, based on physical state and goals^37,71,72^
Unbalanced or suboptimal macro-nutrient distribution (e.g. due to GLP-1 agonist therapy, goals or preferences)	Recommend healthy limits for macros	Overconsumption of energy when consuming too little protein is reported in many species including humans^91^

**Fig. 2:**
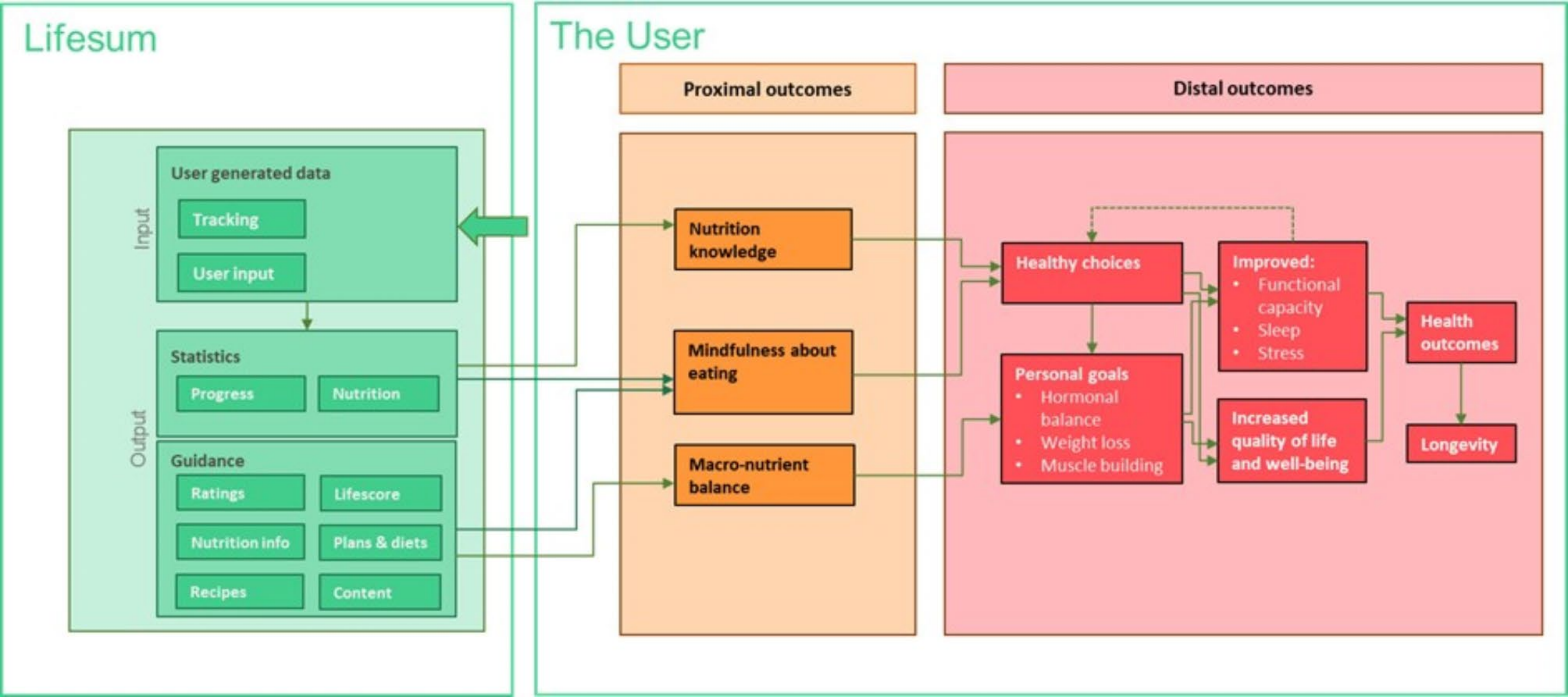
The logic model illustrating the principal functionality of the Lifesum app.

- Implement nutritional information on food items and make food items available to the user easily (e.g. through using a bar code scanner). This will increase nutrition knowledge and lead to healthier choices and also affect habits and perceptions.
- Track daily meals. Achieve this through an easy input of what the user ate and how much. Allow for breakdowns of vegetables, legumes, water etc. if required. This serves to activate and engage the user to achieve mindfulness about eating through self-monitoring and self-regulation.
- Provide feedback and progress. Icon-based food ratings and the ‘Lifescore’ was implemented to achieve this. The Lifescore is a compilation score provided to the user to illustrate the food quality in an understandable way to offer positive reinforcement to limit feelings of deprivation and boost enjoyment with the app user experience.

### Logic model and the Lifesum app

Considering the theoretical model and the formulated intervention theory, the Lifesum app can now be described using a logic model. The intervention theory lets us go back to the theoretical model and refine the proximal outcome “engaging in healthy eating” to be made up of three constructs: nutrition knowledge, mindfulness about eating and macro-nutrient balance. The logic model of the Lifesum app is illustrated in Fig. 2.

The user will provide data about biometrics, goals and preferences, as well as the daily tracking of meals. The Lifesum app will at a high level generate statistics and guidance from inputting the user data into the algorithms of the app. The statistics and guidance fed back to the user will, according to the intervention theory, lead to the proximal outcomes increased nutrition knowledge, mindfulness about eating and the appropriate macro-nutrient balance. The distal outcomes are based on findings from literature, as described in the earlier section of this manuscript.

## Discussion

We have shown how a scientific approach can be combined with the fast-paced development of mobile apps, starting with outlining and describing a theoretical model or framework to guide future decisions. An intervention theory based on published science was constructed, where the most appealing aspects of eating- and diet-related behaviours were targeted and analysed. The theories came together to shape the Lifesum app, and it was functionally described using a logic model.

To use a theoretical model on which to base the design of an intervention, is far superior than the “it seemed like a good idea at the time” (or ISLAGIATT) approach^92^, and is also recommended by the MRC’s guidance on developing complex health interventions^17^. There is an abundance of theoretical models revolving around behaviour change in general, with the most commonly referenced being the Transtheoretical Model of Change, the Theory of Planned Behaviour, Social Cognitive Theory, the Information-Motivation-Behavioural-Skills Model, the Health belief model and self-determination theory^93^ and the as of late more commonly used Behaviour change wheel^94^. Since the field of mobile health in general and mobile apps in particular is such a rapidly changing field^95^, we decided to create our own model in a more pragmatic way, but still leveraging the most important learnings from science on how to introduce, encourage and sustain new behaviours^26^. The model emphasizes two main antecedents to the desired behaviour, namely personal perceptions of the user (and how they will spawn motivation to engage in the desired behaviour), and the equally important time-tools-theory construct. These aspects led to decisions on including information, Lifescore and other ‘feedback loops’ to enable the former, while incorporating ready-made recipes, bar-code scanner and meal plans to enable the latter. These examples serve to illustrate how the underlying theory was used to guide and shape design decisions.

The intervention theory generation is a time-consuming work, involving substantial literature reviews and scrutiny of ideas, where the most important step is to identify relevant factor-action pairs. It is an effective way of assuring that once the design of the intervention starts, there are very good grounds for the approaches taken, and close to 500 peer-reviewed papers have cited this strategy in their work. We identified three pivotal factors, where what we seek to achieve with the Lifesum app is provision of nutritional knowledge, increased mindfulness about eating and the appropriate macro-nutrient balance depending on the users’ goals.

Information is a key-component to drive healthy eating as we have shown in this manuscript. There are two different types of knowledge, namely declarative knowledge – e.g. knowing the fibre content of a fruit or the fat content of whole milk, and then there is procedural knowledge, which could for example be knowing how to choose the healthier of two snacks or how to compose a balanced menu^96^. After knowledge has been obtained, there is also the matter of the decision process regarding foods, which can result from either impulsive or controlled reasoning routes. The more impulsive route is related to implicit attitudes of the individual where decisions happen more or less automatically, in contrast to the explicit attitudes which stem from an active reasoning and a more deliberate and active choice^70^. It has been shown that food purchasing decisions usually happen fast and without deliberation^97^, suggesting that building procedural knowledge through for instance cooking tips, meal suggestions and recipes is a potent way to build the right type of knowledge, and was hence implemented in Lifesum.

Poor adherence to calorie restricted diets has been reported for decades and has been reported to cause eating disorders and ultimately obesity^57,81,82,87^. As previously shown, being aware of what and how you eat is required to combat feelings of deprivation or poor self-regulation. A key to being generally aware of what you eat in a positive way, is to employ self-monitoring for instance through tracking foods in some type of food diary^57,81–83^. Keeping a person actively engaged makes the person highly aware of food intake, leading to a more mindful disposition towards food^56,86^ and developing a more mindful approach to food and eating limits risks of eating disorders^98^. Achieving mindful eating through tracking of foods with all it entails is hence the second of the three main features of Lifesum.

Macro-nutrient balance has profound impacts on a person’s health. There are close to 70 controlled studies that connect sugar consumption (i.e. high-carbohydrate diet) to body-fat^71^ and also scientific consensus to avoid the highly palatable ultra processed foods, i.e. junk food^99^. Humans are not good at controlling daily food intake, but are very good at it over a period of several days^100^, and therefore daily support is desirable. The science is also clear that there doesn’t seem to be a “one size fit all” approach to macro-nutrient balance that is feasible, although certain spans are generally recommended^37,71,89,99,100^. As a consequence, recommendations need to be based on BMI, sex, age and also possibly previous weight loss history, and certainly subjective goals, which could be muscle gain, longevity or weight loss or any combination thereof, as well as possibly also disease history^37,71–74,88,89,101^. Consequently, Lifesum collects most of these parameters and enable these to impact the user’s recommended macro-nutrient distribution, in a way that is different for each user. This macro-nutrient control is the third of the three main features of Lifesum.

In the combined learnings from the theoretical model and the intervention theory, the features of the app crystalize. It is a useful approach to zoom out and mechanistically describe the app (or any other intervention) in a logic model. It also makes for adding future features easier, as inspiration from the theoretical model or the intervention theory will be easy to see how it fits in the existing structure of the app. Furthermore, it is a powerful way to analyse the app for gaps and opportunities.

As shown in the logic model (Fig. 2), the user provides input to the app. In that situation, dietary preferences such as vegan or ketogenic are noted and will guide the recipes suggested and also controls the macros. This way the appropriate macro-nutrient balance is attained, regardless of dietary preferences. The verified food items included in the app and their respective nutritional information have been imported from the United States Department of Agriculture, and “Livsmedelsverket” (the Swedish Food Agency).

This study has limitations in that academic work moves slower than the private sector does, and consequently what is published will inevitably be somewhat out-of-date. Furthermore, these are all theoretical assumptions based on evidence in the literature, and all hypotheses relating to both the proximal and distal outcomes described herein need to be studied in prospective trials before any claims can be made.

While acknowledging these limitations, we believe that this manuscript may serve as inspiration for other organizations working with wellness apps or similar, to spend the time to make efforts largely evidence based. Overall, a greater awareness of the impact of nutrition on different pillars of health such as sleep, ability to focus, and energy levels remains desirable. With the obesity epidemic, increased interest in health and wellness and more recently with the introduction of GLP-1 agonists, nutritional support has become increasingly important for an increasingly large group of people. Since GLP-1 drugs are usually not intended for life-long use, implementing behavior change related to nutrition habits becomes a crucial component to GLP-1 treatment.

We have also shown that despite the wellness app field being a very fast-moving entity, there is still room for science. Future steps include prospective validation studies, where the proposed proximal outcomes are confirmed or rejected. Preliminary data show reductions in BMI and body weight and high retention rates. There are also indications that sleep, and stress parameters improved. All of these possible outcomes however remain to be proven in future studies.

## Conclusions

To conclude, we have developed a theoretical model for behaviour change related to diet and eating. Furthermore, we identified issues pertaining to diets and food, and described how these may be influenced to generate positive effects or outcomes. These combined insights were then used to mechanistically describe the Lifesum app. With this study, and the establishment of the conceptual framework and intervention design, the foundation is laid for future research on effects and outcomes.

## Data Availability

The datasets used and/or analysed during the current study available from the corresponding author on reasonable request.

## Abbreviations

BMI: Body-mass index
GLP-1: Glucagon-like peptide-1
MRC: Medical Research Council

## References

[1] Bart, R. et al. The assessment and measurement of wellness in the clinical medical setting: A systematic review. *Innov. Clin. Neurosci.* **15** (09–10), 14 (2018). PMC6292717 30588362

[2] Callaghan, S., Lösch, M., Pione, A. & Teichner, W. Accessed: 0611. Feeling good: The future of the $1.5 trillion wellness market (2022). https://www mckinseycom/industries/consumer-packaged-goods/our-insights/feeling-good-the-future-of-the-1-5-trillion-wellness-market 2021.

[3] Kauppi, K., Vanhala, A., Roos, E. & Torkki, P. Assessing the structures and domains of wellness models: A systematic review. *Int. J. Wellbeing*. **13** (2), 1–19 (2023).

[4] Peñalvo, J. L. et al. Effectiveness of workplace wellness programmes for dietary habits, overweight, and cardiometabolic health: A systematic review and meta-analysis. *Lancet Public. Health*. **6** (9), e648–e60 (2021). 34454642 10.1016/S2468-2667(21)00140-7 PMC8627548

[5] Gao, Y., Lopez, R. A., Liao, R. & Liu, X. Public health shocks, learning and diet improvement. *Food Policy*. **112**, 102365 (2022). 36267324 10.1016/j.foodpol.2022.102365 PMC9559314

[6] Spronk, I., Kullen, C., Burdon, C. & O’Connor, H. Relationship between nutrition knowledge and dietary intake. *Br. J. Nutr.* **111** (10), 1713–1726 (2014). 24621991 10.1017/S0007114514000087

[7] Kim, J. Y. Optimal diet strategies for weight loss and weight loss maintenance. *J. Obes. Metabolic Syndrome*. **30** (1), 20 (2021). 10.7570/jomes20065 PMC8017325 33107442

[8] Wasil, A. R., Palermo, E., Lorenzo-Luaces, L. & DeRubeis, R. Is there an app for that? A review of popular mental health and wellness apps. (2021).

[9] Gordon, W. J., Landman, A., Zhang, H. & Bates, D. W. Beyond validation: Getting health apps into clinical practice. *NPJ Digit. Med.* **3** (1), 14 (2020). 32047860 10.1038/s41746-019-0212-z PMC6997363

[10] WHO. mHealth: new horizons for health through mobile technologies. (2011).

[11] McKay, F. H. et al. Evaluating mobile phone applications for health behaviour change: A systematic review. *J. Telemed. Telecare*. **24** (1), 22–30 (2018). 27760883 10.1177/1357633X16673538

[12] Milne-Ives, M., Lam, C., De Cock, C., Van Velthoven, M. H. & Meinert, E. Mobile apps for health behavior change in physical activity, diet, drug and alcohol use, and mental health: Systematic review. *JMIR mHealth uHealth*. **8** (3), e17046 (2020). 32186518 10.2196/17046 PMC7113799

[13] König, L. M., Attig, C., Franke, T. & Renner, B. Barriers to and facilitators for using nutrition apps: Systematic review and conceptual framework. *JMIR mHealth uHealth*. **9** (6), e20037 (2021). 34254938 10.2196/20037 PMC8409150

[14] De Croon, R., Geuens, J., Verbert, K. & Vanden Abeele, V. (eds) A systematic review of the effect of gamification on adherence across disciplines. International conference on human-computer interaction (Springer, 2021).

[15] Eikey, E. V. Effects of diet and fitness apps on eating disorder behaviours: Qualitative study. *BJPsych Open.* **7** (5), e176 (2021).

[16] Tran, S., Smith, L., El-Den, S. & Carter, S. The use of gamification and incentives in mobile health apps to improve medication adherence: Scoping review. *JMIR mHealth uHealth*. **10** (2), e30671 (2022). 35188475 10.2196/30671 PMC8902658

[17] Skivington, K. et al. A new framework for developing and evaluating complex interventions: Update of medical research Council guidance. *Bmj* **374**. (2021). 10.1136/bmj.n2061 PMC8482308 34593508

[18] Ryan, P. Integrated theory of health behavior change: Background and intervention development. *Clin. Nurse Spec.* **23** (3), 161–170 (2009). 19395894 10.1097/NUR.0b013e3181a42373 PMC2778019

[19] Moullin, J. C. et al. Ten recommendations for using implementation frameworks in research and practice. *Implement. Sci. Commun.* **1**, 1–12 (2020). 32885199 10.1186/s43058-020-00023-7 PMC7427911

[20] Toomey, E. et al. Focusing on fidelity: Narrative review and recommendations for improving intervention fidelity within trials of health behaviour change interventions. *Health Psychol. Behav. Med.* **8** (1), 132–151 (2020). 34040865 10.1080/21642850.2020.1738935 PMC8114368

[21] Bennett, J. A. Mediator and moderator variables in nursing research: Conceptual and statistical differences. *Res. Nurs. Health*. **23** (5), 415–420 (2000). 11052395 10.1002/1098-240x(200010)23:5<415::aid-nur8>3.0.co;2-h

[22] Moore, G. F. et al. Process evaluation of complex interventions: Medical research Council guidance. *Bmj* **350**. (2015). 10.1136/bmj.h1258 PMC4366184 25791983

[23] Movsisyan, A. et al. Adapting evidence-informed complex population health interventions for new contexts: A systematic review of guidance. *Implement. Sci.* **14**, 1–20 (2019). 31847920 10.1186/s13012-019-0956-5 PMC6918624

[24] Wight, D., Wimbush, E., Jepson, R. & Doi, L. Six steps in quality intervention development (6SQuID). *J. Epidemiol. Commun. Health*. **70** (5), 520–525 (2016). 10.1136/jech-2015-205952 PMC4853546 26573236

[25] Foundation, W. K. *WK Kellogg Foundation Logic Model Development Guide* (WK Kellogg Foundation, 2004).

[26] Kwasnicka, D., Dombrowski, S. U., White, M. & Sniehotta, F. Theoretical explanations for maintenance of behaviour change: A systematic review of behaviour theories. *Health Psychol. Rev.* **10** (3), 277–296 (2016). 26854092 10.1080/17437199.2016.1151372 PMC4975085

[27] Fitzgerald, M. & McClelland, T. What makes a mobile app successful in supporting health behaviour change? *Health Educ. J.* **76** (3), 373–381 (2016).

[28] Collins, L. M., Murphy, S. A., Nair, V. N. & Strecher, V. J. J. A. B. M. A strategy for optimizing and evaluating behavioral interventions. **30** (1), 65–73. (2005). 10.1207/s15324796abm3001_8 16097907

[29] Cane, J., O’Connor, D. & Michie, S. Validation of the theoretical domains framework for use in behaviour change and implementation research. *Implement. Sci.* **7**, 1–17 (2012). 10.1186/1748-5908-7-37 PMC3483008 22530986

[30] Trindade, F. Nutritional influences on hormonal health. Integrative and Functional Medical Nutrition Therapy: Principles and Practices. 517–32. (2020).

[31] Schwarz, N. A., Rigby, B. R., La Bounty, P., Shelmadine, B. & Bowden, R. G. A review of weight control strategies and their effects on the regulation of hormonal balance. *J. Nutr. Metabol*. **2011** (1), 237932 (2011). 10.1155/2011/237932 PMC3147122 21822485

[32] Abete, I., Astrup, A., Martínez, J. A., Thorsdottir, I. & Zulet, M. A. Obesity and the metabolic syndrome: Role of different dietary macronutrient distribution patterns and specific nutritional components on weight loss and maintenance. *Nutr. Rev.* **68** (4), 214–231 (2010). 20416018 10.1111/j.1753-4887.2010.00280.x

[33] Lofgren, I. E. Mindful eating: An emerging approach for healthy weight management. *Am. J. Lifestyle Med.* **9** (3), 212–216 (2015).

[34] Bray, G. A. & Siri-Tarino, P. W. The role of macronutrient content in the diet for weight management. *Endocrinol. Metabol. Clin.* **45** (3), 581–604 (2016). 10.1016/j.ecl.2016.04.009 27519132

[35] Rolls, B. J., Drewnowski, A. & Ledikwe, J. H. Changing the energy density of the diet as a strategy for weight management. *J. Am. Diet. Assoc.* **105** (5), 98–103 (2005). 15867904 10.1016/j.jada.2005.02.033

[36] Stokes, T., Hector, A. J., Morton, R. W., McGlory, C. & Phillips, S. M. Recent perspectives regarding the role of dietary protein for the promotion of muscle hypertrophy with resistance exercise training. *Nutrients* **10** (2), 180 (2018). 29414855 10.3390/nu10020180 PMC5852756

[37] Schoenfeld, B. J. & Aragon, A. A. How much protein can the body use in a single meal for muscle-building? Implications for daily protein distribution. *J. Int. Soc. Sports Nutr.* **15** (1), 10 (2018). 29497353 10.1186/s12970-018-0215-1 PMC5828430

[38] Morton, K. et al. Using digital interventions for self-management of chronic physical health conditions: A meta-ethnography review of published studies. *Patient Educ. Couns.* **100** (4), 616–635 (2017). 28029572 10.1016/j.pec.2016.10.019 PMC5380218

[39] Galbreath, M. et al. Effects of adherence to a higher protein diet on weight loss, markers of health, and functional capacity in older women participating in a resistance-based exercise program. *Nutrients* **10** (8), 1070 (2018). 30103509 10.3390/nu10081070 PMC6115985

[40] Kumagai, S. et al. Effects of dietary variety on declines in high-level functional capacity in elderly people living in a community. [Nihon koshu eisei zasshi]. *Japanese J. Public. Health*. **50** (12), 1117–1124 (2003). 14750363

[41] Bales, C. W. & Starr, K. N. P. Obesity interventions for older adults: Diet as a determinant of physical function. *Adv. Nutr.* **9** (2), 151–159 (2018). 29659687 10.1093/advances/nmx016 PMC5916429

[42] St-Onge, M-P., Mikic, A. & Pietrolungo, C. E. Effects of diet on sleep quality. *Adv. Nutr.* **7** (5), 938–949 (2016). 27633109 10.3945/an.116.012336 PMC5015038

[43] Binks, H., Vincent, E., Gupta, G., Irwin, C. & Khalesi, C. Effects of diet on sleep: A narrative review. *Nutrients* **12** (4), 936 (2020). 32230944 10.3390/nu12040936 PMC7230229

[44] Godos, J. et al. Association between diet and sleep quality: A systematic review. *Sleep Med. Rev.* **57**, 101430 (2021). 33549913 10.1016/j.smrv.2021.101430

[45] Bremner, J. D. et al. Diet, stress and mental health. *Nutrients* **12** (8), 2428 (2020). 32823562 10.3390/nu12082428 PMC7468813

[46] Godos, J. et al. Diet and mental health: Review of the recent updates on molecular mechanisms. *Antioxidants* **9** (4), 346 (2020). 32340112 10.3390/antiox9040346 PMC7222344

[47] Stachowicz, M. & Lebiedzińska, A. The effect of diet components on the level of cortisol. *Eur. Food Res. Technol.* **242**, 2001–2009 (2016).

[48] Wardle, J., Parmenter, K. & Waller, J. Nutrition knowledge and food intake. *Appetite* **34** (3), 269–275 (2000). 10888290 10.1006/appe.1999.0311

[49] Hendrie, G., Cox, D. & Coveney, J. P17: Nutrition knowledge as a predictor of nutrient intake and diet quality. *J. Nutr. Educ. Behav.* **40** (4), S49–S50 (2008).

[50] Barreiro-Hurlé, J., Gracia, A. & De-Magistris, T. Does nutrition information on food products lead to healthier food choices? *Food Policy*. **35** (3), 221–229 (2010).

[51] Milosavljević, D., Mandić, M. L. & Banjari, I. Nutritional knowledge and dietary habits survey in high school population. *Coll. Antropol.* **39** (1), 101–107 (2015). 26040077

[52] Kullen, C. J., Farrugia, J-L., Prvan, T. & O’Connor, H. T. Relationship between general nutrition knowledge and diet quality in Australian military personnel. *Br. J. Nutr.* **115** (8), 1489–1497 (2016). 26931550 10.1017/S0007114516000532

[53] Akkartal, Ş. & Gezer, C. Is nutrition knowledge related to diet quality and obesity? *Ecol. Food Nutr.* **59** (2), 119–129 (2020). 31590573 10.1080/03670244.2019.1675654

[54] Geaney, F. et al. Nutrition knowledge, diet quality and hypertension in a working population. *Prev. Med. Rep.* **2**, 105–113 (2015). 26844058 10.1016/j.pmedr.2014.11.008 PMC4721350

[55] Ferreira-Pêgo, C., Rodrigues, J., Costa, A. & Sousa, B. Eating behavior: The influence of age, nutrition knowledge, and mediterranean diet. *Nutr. Health*. **26** (4), 303–309 (2020). 32779518 10.1177/0260106020945076

[56] Ouwens, M., Schiffer, A., Visser, L., Raeijmaekers, N. & Nyklíček, I. Mindfulness and eating behaviour styles in morbidly obese males and females. *Appetite* **87**, 62–67 (2015). 25478687 10.1016/j.appet.2014.11.030

[57] Donofry, S. D. et al. Effect of dietary restraint and mood state on attentional processing of food cues. *J. Behav. Ther. Exp. Psychiatry*. **62**, 117–124 (2019). 30316044 10.1016/j.jbtep.2018.10.002

[58] Keski-Rahkonen, A. Epidemiology of binge eating disorder: Prevalence, course, comorbidity, and risk factors. *Curr. Opin. Psychiatry*. **34** (6), 525–531 (2021). 34494972 10.1097/YCO.0000000000000750

[59] Qian, J. et al. An update on the prevalence of eating disorders in the general population: A systematic review and meta-analysis. Eating and Weight Disorders-Studies on Anorexia, Bulimia and Obesity. 1–14. (2021). 10.1007/s40519-021-01162-z PMC8933366 33834377

[60] Santomauro, D. F. et al. The hidden burden of eating disorders: An extension of estimates from the global burden of disease study 2019. *Lancet Psychiatry*. **8** (4), 320–328 (2021). 33675688 10.1016/S2215-0366(21)00040-7 PMC7973414

[61] Gralle, A. P. B. P. et al. Job strain and binge eating among Brazilian workers participating in the ELSA-Brasil study: Does BMI matter? *J. Occup. Health*. **59** (3), 247–255 (2017). 28163281 10.1539/joh.16-0157-OA PMC5478507

[62] Leung, S. L., Barber, J. A., Burger, A. & Barnes, R. D. Factors associated with healthy and unhealthy workplace eating behaviours in individuals with overweight/obesity with and without binge eating disorder. *Obes. Sci. Pract.* **4** (2), 109–118 (2018). 29670748 10.1002/osp4.151 PMC5893464

[63] Brown, S. et al. A qualitative exploration of the impact of COVID-19 on individuals with eating disorders in the UK. *Appetite* **156**, 104977 (2021). 32991945 10.1016/j.appet.2020.104977 PMC7521890

[64] Rodgers, R. F. et al. The impact of the COVID-19 pandemic on eating disorder risk and symptoms. *Int. J. Eat. Disord.* **53** (7), 1166–1170 (2020). 32476175 10.1002/eat.23318 PMC7300468

[65] Vuillier, L., May, L., Greville-Harris, M., Surman, R. & Moseley, R. L. The impact of the COVID-19 pandemic on individuals with eating disorders: The role of emotion regulation and exploration of online treatment experiences. *J. Eat. Disorders*. **9**, 1–18 (2021). 10.1186/s40337-020-00362-9 PMC7802411 33436064

[66] Peleg, O., Idan, M. & Katz, R. Exploring the relationship between binge eating and differentiation of self: The mediating role of emotional distress and work stress. *Front. Nutr.* ;**11**. (2024). 10.3389/fnut.2024.1368995 PMC11260811 39040923

[67] Jordan, C. H., Wang, W., Donatoni, L. & Meier, B. P. Mindful eating: Trait and state mindfulness predict healthier eating behavior. *Pers. Indiv. Differ.* **68**, 107–111 (2014).

[68] Monroe, J. T. Mindful eating: Principles and practice. *Am. J. Lifestyle Med.* **9** (3), 217–220 (2015).

[69] Bublitz, M. G., Peracchio, L. A. & Block, L. G. Why did I eat that? Perspectives on food decision making and dietary restraint. *J. Consumer Psychol.* **20** (3), 239–258 (2010).

[70] Demartini, E. et al. Changing attitudes towards healthy food via self-association or nutritional information: What works best? *Appetite* **132**, 166–174 (2019). 30119922 10.1016/j.appet.2018.08.001

[71] Martinez, J. A., Navas-Carretero, S., Saris, W. H. & Astrup, A. Personalized weight loss strategies—the role of macronutrient distribution. *Nat. Rev. Endocrinol.* **10** (12), 749–760 (2014). 10.1038/nrendo.2014.175 25311395

[72] Adamska-Patruno, E. et al. The relationship between the Leptin/ghrelin ratio and meals with various macronutrient contents in men with different nutritional status: A randomized crossover study. *Nutr. J.* **17** (1), 1–7 (2018). 30593267 10.1186/s12937-018-0427-x PMC6309055

[73] Bosy-Westphal, A., Hägele, F. A. & Müller, M. J. Impact of energy turnover on the regulation of energy and macronutrient balance. *Obesity* **29** (7), 1114–1119 (2021). 34002543 10.1002/oby.23133

[74] Kitada, M., Ogura, Y., Monno, I. & Koya, D. The impact of dietary protein intake on longevity and metabolic health. *EBioMedicine* **43**, 632–640 (2019). 30975545 10.1016/j.ebiom.2019.04.005 PMC6562018

[75] Solon-Biet, S. M. et al. The ratio of macronutrients, not caloric intake, dictates cardiometabolic health, aging, and longevity in ad libitum-fed mice. *Cell Metabol.* **19** (3), 418–430 (2014). 10.1016/j.cmet.2014.02.009 PMC5087279 24606899

[76] Näslund, E. et al. Energy intake and appetite are suppressed by glucagon-like peptide-1 (GLP-1) in obese men. *Int. J. Obes.* **23** (3), 304–311 (1999). 10.1038/sj.ijo.0800818 10193877

[77] Christensen, S., Robinson, K., Thomas, S. & Williams, D. R. Dietary intake by patients taking GLP-1 and dual GIP/GLP-1 receptor agonists: A narrative review and discussion of research needs. *Obes. Pillars* :100121. (2024). 10.1016/j.obpill.2024.100121 PMC11340591 39175746

[78] Gorgojo-Martínez, J. J. et al. Clinical recommendations to manage Gastrointestinal adverse events in patients treated with Glp-1 receptor agonists: a multidisciplinary expert consensus. *J. Clin. Med.* **12** (1), 145 (2022). 36614945 10.3390/jcm12010145 PMC9821052

[79] Worsley, A. Nutrition knowledge and food consumption: Can nutrition knowledge change food behaviour? *Asia Pac. J. Clin. Nutr.* **11**, S579–S85 (2002). 12492651 10.1046/j.1440-6047.11.supp3.7.x

[80] O’Brien, G. & Davies, M. Nutrition knowledge and body mass index. *Health Educ. Res.* **22** (4), 571–575 (2007). 17041019 10.1093/her/cyl119

[81] Ruderman, A. J. Dietary restraint: A theoretical and empirical review. *Psychol. Bull.* **99** (2), 247 (1986). 3515384

[82] Schaumberg, K., Anderson, D., Anderson, L., Reilly, E. & Gorrell, S. Dietary restraint: what’s the harm? A review of the relationship between dietary restraint, weight trajectory and the development of eating pathology. *Clin. Obes.* **6** (2), 89–100 (2016). 26841705 10.1111/cob.12134

[83] Johnson, F., Pratt, M. & Wardle, J. Dietary restraint and self-regulation in eating behavior. *Int. J. Obes.* **36** (5), 665–674 (2012). 10.1038/ijo.2011.156 21829162

[84] Kristeller, J., Wolever, R. Q. & Sheets, V. Mindfulness-based eating awareness training (MB-EAT) for binge eating: A randomized clinical trial. *Mindfulness* **5**, 282–297 (2014).

[85] Warren, J. M., Smith, N. & Ashwell, M. A structured literature review on the role of mindfulness, mindful eating and intuitive eating in changing eating behaviours: Effectiveness and associated potential mechanisms. *Nutr. Res. Rev.* **30** (2), 272–283 (2017). 28718396 10.1017/S0954422417000154

[86] Sala, M., Shankar Ram, S., Vanzhula, I. A. & Levinson, C. A. Mindfulness and eating disorder psychopathology: A meta-analysis. *Int. J. Eat. Disord.* **53** (6), 834–851 (2020). 32100320 10.1002/eat.23247

[87] Middlemass, K. M. et al. Food insecurity & dietary restraint in a diverse urban population. *Eat. Disord.* **29** (6), 616–629 (2021). 32129723 10.1080/10640266.2020.1723343

[88] Bellissimo, N. & Akhavan, T. Effect of macronutrient composition on short-term food intake and weight loss. *Adv. Nutr.* **6** (3), 302S–8S (2015). 25979503 10.3945/an.114.006957 PMC4424768

[89] Acheson, K. Diets for body weight control and health: The potential of changing the macronutrient composition. *Eur. J. Clin. Nutr.* **67** (5), 462–466 (2013). 23187953 10.1038/ejcn.2012.194

[90] Samoggia, A. & Riedel, B. Assessment of nutrition-focused mobile apps’ influence on consumers’ healthy food behaviour and nutrition knowledge. *Food Res. Int.* **128**, 108766 (2020). 31955740 10.1016/j.foodres.2019.108766

[91] Simpson, S. J. & Raubenheimer, D. Macronutrient balance and lifespan. *Aging (Albany NY)*. **1** (10), 875 (2009). 20157561 10.18632/aging.100098 PMC2815731

[92] D’Lima, D., Lorencatto, F. & Michie, S. *The Behaviour Change Wheel Approach* p. 168–214 (Edward Elgar Publishing, 2020).

[93] Davis, R., Campbell, R., Hildon, Z., Hobbs, L. & Michie, S. Theories of behaviour and behaviour change across the social and behavioural sciences: A scoping review. *Health Psychol. Rev.* **9** (3), 323–344 (2015). 25104107 10.1080/17437199.2014.941722 PMC4566873

[94] Michie, S., van Stralen, M. M. & West, R. The behaviour change wheel: A new method for characterising and designing behaviour change interventions. *Implement. Sci.* **6** (1), 42–53 (2011). 21513547 10.1186/1748-5908-6-42 PMC3096582

[95] Ben-Zeev, D. et al. Strategies for mHealth research: Lessons from 3 mobile intervention studies. *Adm. Policy Mental Health Mental Health Serv. Res.* **42** (2), 157–167 (2015). 10.1007/s10488-014-0556-2 PMC4232479 24824311

[96] Dickson-Spillmann, M. & Siegrist, M. Consumers’ knowledge of healthy diets and its correlation with dietary behaviour. *J. Hum. Nutr. Dietetics*. **24** (1), 54–60 (2011). 10.1111/j.1365-277X.2010.01124.x 20880377

[97] Grunert, K. G., Wills, J. M. & Fernández-Celemín, L. Nutrition knowledge, and use and Understanding of nutrition information on food labels among consumers in the UK. *Appetite* **55** (2), 177–189 (2010). 20546813 10.1016/j.appet.2010.05.045

[98] Christodoulou, E., Markopoulou, V. & Koutelidakis, A. E. Exploring the link between mindful eating, Instagram engagement, and eating disorders: A focus on orthorexia nervosa. *Psychiatry Int.* **5** (1), 27–38 (2024).

[99] Schoeller, D. A. & Buchholz, A. C. Energetics of obesity and weight control: Does diet composition matter? *J. Am. Diet. Assoc.* **105** (5), 24–28 (2005). 10.1016/j.jada.2005.02.025 15867892

[100] Flatt, J. P. Macronutrient composition and food selection. *Obes. Res.* **9** (S11), 256S–62S (2001). 11707551 10.1038/oby.2001.128

[101] Wadden, T. A., Butryn, M. L. & Byrne, K. J. Efficacy of lifestyle modification for long-term weight control. *Obes. Res.* **12** (S12), 151S–62S (2004). 15687411 10.1038/oby.2004.282

